# Identification of a phenoloxidase- and melanin-dependent defence mechanism in *Achatina fulica* infected with *Angiostrongylus vasorum*

**DOI:** 10.1186/s13071-018-2710-2

**Published:** 2018-02-27

**Authors:** Aytube Lucas Coaglio, Mônica Alves Neves Diniz Ferreira, Walter dos Santos Lima, Cíntia Aparecida de Jesus Pereira

**Affiliations:** 1 Departamento de Parasitologia, Instituto de Ciências Biológicas, Universidade Federal de Minas Gerais, Belo Horizonte, Brazil; 2 Departamento de Patologia Geral, Instituto de Ciências Biológicas, Universidade Federal de Minas Gerais, Belo Horizonte, Brazil

**Keywords:** *Angiostrongylus vasorum*, *Achatina fulica*, Phenoloxidase, Melanin, Innate immune defence

## Abstract

**Background:**

*Angiostrongylus vasorum* has different freshwater aquatic and terrestrial gastropod molluscs as an intermediate host, e.g. *Arion* spp. The mollusc *Achatina fulica* is a danger to public health, given the large diversity of nematodes utilizing it as an intermediate host, such as the parasites of the genus *Angiostrongylus*, of importance in human and veterinary medicine. *Achatina fulica* has been shown to have an excellent capacity for maintaining outbreaks and natural infections with *A. cantonensis* in Asia. Within the mollusc, the nematode parasites activate haemocytes and/or haemolymph factors and in some invertebrates, phenoloxidase (PO), that induces the release of toxic elements and eliminates the parasites. Despite the importance of *A. fulica* in the life-cycle of nematodes, little is known regarding the defence mechanisms involving PO in molluscs infected with nematodes. Here, the presence of PO and nitric oxide (NO) in the haemolymph and haemocytes of *A. fulica* infected with first-stage (L1) larvae of *Angiostrongylus vasorum* was evaluated, together with the presence of melanin in the cephalopod mollusc tissue.

**Results:**

An increase in PO at one day post infection (dpi), in comparison with the control using the substrates L-tyrosine (*F*_(4,90)_ = 6.73, *P* = 0.00006), L-DOPA (*F*_(4,90)_ = 22.67, *P* = 0.02) and p-phenylenediamine (PPD) (*F*_(4,90)_ = 27.58, *P* = 0.0019), was observed. PO increase coincided with the presence of melanin in the cephalopodal tissue. At 8 dpi, PO activity, compared to L-DOPA (*F*_(4,90)_ = 22.67, *P* = 0.00002) and PPD (*F*_(4,90)_ = 27.58, *P* = 0.079) decreased, while melanin increased. At 13 dpi, PO decreased with PPD (*F*_(4,90)_ = 27.58, *P* = 0.000015) and also the amount of melanin observed in histology. At 30 dpi, PO increased along with the substrates L-DOPA and PPD, while melanin decreased. NO levels increased until 8 dpi, and decreased after 13 dpi.

**Conclusions:**

To our knowledge, this is the first study that illustrates PO activity in a helminth-infected *A. fulica* and provides the first observation of an L-tyrosine dependent PO activity in molluscs infected with *A. vasorum*. This work suggests that PO pathway may help to control *A. vasorum* infection in *A. fulica*.

## Background

*Angiostrongylus vasorum* parasitizes pulmonary arteries in domestic and wild Canidae and has terrestrial molluscs as intermediate hosts [1–6]. The molluscs are infected with first-stage (L1) larvae eliminated via the dogs’ faeces. Inside the mollusc, L1 go through two moults to become second- (L2) and third-stage (L3) larvae, the latter being infective to the definitive hosts [7].

The mollusc intermediate host is capable of mounting a cellular immune response to parasite infection via factors soluble in the haemolymph that activate haemocytes. In addition, elements of the acellular fraction of the haemolymph can also damage the parasites’ structures [8, 9]. In some bivalves, following the recognition and encapsulation of the pathogens, the phenoloxidase (PO) pathway responsible for melanisation is activated, as well as phagocytosis and nodulation, which can sometimes result in parasite elimination [10].

PO exists in the haemolymph in its inactive form (proPO) and is activated by external components of microorganisms such as β-1,3-glucans [11] and lipopolysaccharides (LPS) [12]. These compounds induce the cleavage of proPO into PO by endogenous serine proteases [13]. Then, PO catalyses the hydroxylation of monophenols such as the amino acid tyrosine to o-diphenol (DOPA or DOPAmine), followed by their oxidation to o-quinones (DOPAquinone and DOPAminequinone) [14, 15]. At the end of the cascade, the dark pigment melanin is formed, thereby contributing to the asphyxiation of the pathogen [16].

POs are copper-binding proteins including tyrosinases (EC 1.14.18.1), catecholases (EC 1.10.3.1), and laccases (EC 1.10.3.2), and play a vital role in the immunological defence mechanism of Pacific oyster [17]. All the three types of PO can oxidise o-diphenols, such as L-3,4-dihydroxyphenylalanine (L-DOPA, catecholase activity). However, only the tyrosinases can hydroxylate monophenols such as L-tyrosine (monophenolase activity), and only laccases can oxidise m- and p-diphenols, or aromatic compounds containing amine groups such as p-phenylenediamine (PPD; laccase activity) [17, 18].

Melanin deposition around the pathogens, or in the pathogens themselves, is formed during the oxidation and polymerisation of phenols. The cytotoxic molecules generated in this process include reactive oxygen (ROS) and nitrogen (RNS) species and quinoids, which are intermediates of melanin. Additionally, an increase in the production of nitric oxide (NO), an effector molecule very effective against invasive organisms, occurs in immunoreactive hosts [19, 20].

Despite its importance as an intermediate host of *Angiostrongylus cantonensis* in areas endemic for angiostrongylosis such as Asia and South America [21–26], little is known regarding the innate immune system of *A. fulica*. The role of the PO pathway in controlling infection by nematode parasites is still poorly understood in molluscs and elucidating this process is crucial for understanding the defence mechanisms present in this intermediary host. *Achatina fulica* is less susceptible to *A. vasorum* in comparison with other molluscs and can be infected in laboratory conditions [6]. Here, we used *A. fulica* infected with *A. vasorum* to measure NO levels and characterise PO activity in the soluble fractions of the haemolymph using the substrates L-tyrosine, L-DOPA, and PPD. Additionally, we verified the occurrence of melanin in the cephalopodal tissue of *A. fulica.*

## Methods

### Origin of the parasites and molluscs

A total of 240 specimens of *A. fulica* with an average shell length of 30 mm bred in the Veterinary Helminthology Laboratory of the Federal University of Minas Gerais (LabHelVet-ICB-UFMG) were individually infected with 1000 L1 of *A. vasorum* obtained from dog faeces using the Baermann technique [2]. The strain used was first isolated from a dog in Caratinga, Minas Gerais, by Lima et al. [27].

L1 were added to 2 ml of tap water and placed in a polystyrene container (4 × 5 cm). The molluscs were individually immersed in the suspension for 24 h. The container was sealed with gauze and adhesive tape. Afterwards, the molluscs were transferred to a maintenance tank containing lettuce, water, and autoclaved soil. The L1 that remained in the polystyrene containers were counted to estimate the infection rate [8].

### Collection of haemolymph

The haemolymph was collected according to the changes of stages of *A. vasorum* in *A. fulica*; these stages changes are described according to [6]. The days post infection (dpi) used in the experiment for recovering *A. vasorum* larval stages were 1, 8, 13 and 30 dpi, for recovering L1, L1 to L2, L2 to L3 and L3 stage, respectively. The larval stages were identified using the descriptions of Bessa et al. [28], Barçante et al. [2] and Coaglio et al. [6].

For removal of haemolymph, the shell was disinfected with 70% alcohol and dried on absorbent paper. Then, in the heart region, the shell was pierced with a mini drill (Sigma-Aldrich, Darmstadt, Germany), and the haemolymph was collected by cardiac puncture with a 1000 μl insulin syringe (Manoject 50 unit) and transferred for a 12 ml polypropylene plastic tube in an ice bath. Approximately 200 μl of haemolymph was collected per mollusc and added to the pool of the group, keeping the control and infected pools separated. Samples were collected from 20 molluscs per group, 10 controls and 10 infected, for each day analyzed. The experiments were repeated three times with other snails of the same lineage.

Initially, pH and osmolarity of *A. fulica* haemolymph in the control group were evaluated to analyze the best haemolymph-like buffer solution, which would allow the study of the internal defence system in *A. fulica*. It was noted that pH and osmolarity of haemolymph were pH 7.2 and 160 mOsm/kg, respectively. Thereafter, PBS solution was standardised with the osmolarity and pH of *A. fulica* (NaCl at 71 mM, KCL at 2.7 mM, KH_2_PO_4_ at 1.4 mM, and Na_2_HPO_4_ at 4.3 mM, pH 7.2).

The total haemolymph of each group was centrifuged at 80× *g* for 10 min at 4 °C in a 15 ml polystyrene vial. The supernatant (acellular fraction) was used for the analysis of PO and NO concentration. The haemocytes (cellular fraction) were washed 4× with PBS by centrifugation at 80× *g* for 10 min at 4 °C. The viability of the cells was observed by counting in a hemocytometer after a 1:10 dilution in 0.4% trypan blue (Sigma-Aldrich) [9]. The haemocytes were transferred to 12 ml polypropylene tubes, kept on ice and sonicated for cell lysis. We used three cycles of sonication for 30 s each, with a one-minute interval on ice between the cycles. The haemocyte lysate was centrifuged at 1500× *g* for 10 min at 4 °C, and the supernatant kept on ice for analysis of PO.

### Enzymatic activity of phenoloxidase

Both the acellular fraction and the haemocyte lysate were subjected to a similar analysis of PO. Three substrates specific for PO enzymes were tested: (i) L-tyrosine (monophenol; Sigma-Aldrich) metabolized by tyrosinase only; (ii) L-DOPA (o-diphenol; Sigma-Aldrich) metabolized by the enzymes catecholase, tyrosinase and laccase; and (iii) p-phenylenediamine (PPD; p-diamine; Sigma-Aldrich) metabolized by laccase only [15].

We used 10 mM of L-DOPA (3-4-dihydroxy-L-phenyl-alkaline) (D9628, Sigma-Aldrich), L-tyrosine (CAS number 60-18-4, Sigma-Aldrich), and PPD (CAS number 106-50-3, Sigma-Aldrich) diluted in PBS immediately before the experiment to avoid oxidation.

One-hundred microlitres of cell-free haemolymph, 50 μl of substrate solution, and 50 μl of PBS (total volume of 200 μl) were added to each well into a 96-well microtiter plate. As controls, we used 50 μl of substrate solution with 150 μl PBS. For each treatment, we tested samples, controls, and blanks (200 μl of PBS) in triplicate.

The reaction kinetics were measured for 2 h. Readings were performed every 20 s at 28 °C in a microplate reader (VersaMax Tunable Microplate reader, Molecular Devices, Sunnyvale, CA), using λ = 490 nm for L-tyrosine and L-DOPA, and λ = 465 nm for PPD. The results were plotted using the maximum reaction speed (i.e. the increase in the absorbance/min), according to Alves et al. [29].

### NO dosage in *Achatina fulica*

NO dosage in the acellular fraction of *A. fulica* was determined by measuring the concentration of nitrite (NO_2_) using the Griess test. Fifty microliter aliquots of the samples were allocated to a 96-well plate (Falcon®, BD lab., New York, USA) and 50 μl of Griess reagent were added to each well. Following 10 min of reaction at room temperature, the reading was performed in a spectrophotometer at λ = 570 nm.

### Histology and parasitic load

Histological techniques also analyzed each *A. fulica* specimen used for haemolymph extraction. A 2 cm tissue fragment was separated from the shell and fixed in Milloning [30]. Tissues were dehydrated through an alcohol series, cleared with xylol, and embedded in paraffin [31]. Histological sections of 5 μm thickness were mounted on histological slides and stained with haematoxylin-eosin [32] and Fontana-Masson [33] and then examined using an Olympus BX41 microscope coupled to an Olympus DP12 digital camera. The remaining mollusc tissue was digested with 1% HCl and pepsin (VETEC, 100 units/ml) at 37 °C to estimate the parasite load [6].

### Statistical analysis

PO activity and NO production were analyzed by a one-way ANOVA, followed by the Tukey’s *post-hoc* test. Differences at *P* < 0.05 were considered statistically significant.

## Results

### Parasite load

The average proportion of L1 remaining in the containers used for infection was 25%. Thus, the average number of L1 that penetrated the molluscs was 750. Following the collection of haemolymph and digestion with 1% HCl-pepsin, we recovered larvae from all the molluscs. Percentages varied from 8 to 25% for L1, 7–19% for L2, and 12–30% for L3. Throughout the experiment, no mortality of *A. fulica* was observed.

### Phenoloxidase activity

**Fig. 1:**
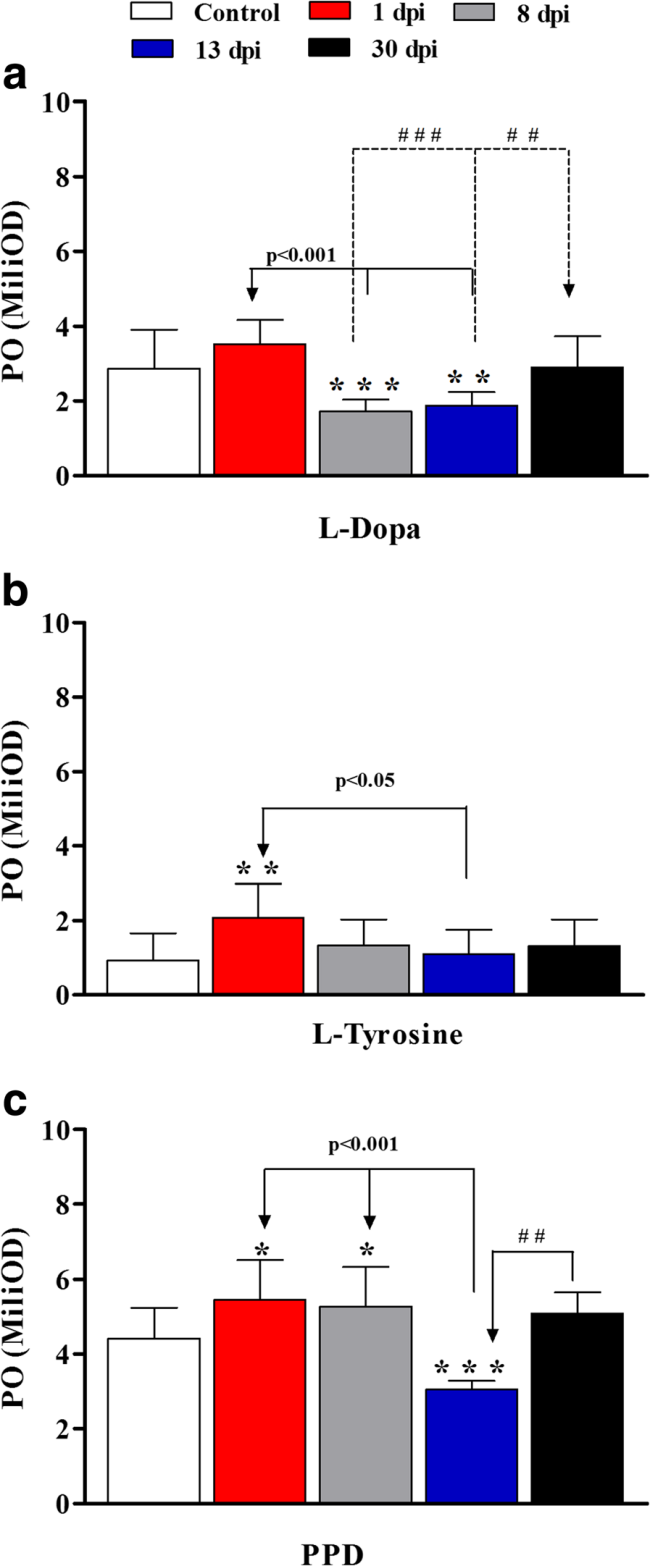
Phenoloxidase (PO) activity in *Achatina fulica* infected with 750 first-stage (L1) larvae of *Angiostrongylus vasorum*, tested with the substrates L-DOPA L-Tyrosine and p- phenylenediamine (PPD). Each *column* represents mean and standard deviation of PO. **a** Analysis with the substrate L-DOPA: **P < 0.001 and *P < 0.01 statistical significance of 8 dpi and 13 dpi, respectively, in comparison with the control; solid arrow (*P* < 0.001), 8 dpi and 13 dpi in comparison with 1 dpi; dashed arrow ### (*P* < 0.001), 30 dpi in comparison with 8 dpi; and ## (*P* < 0.01), 30 dpi in comparison with 13 dpi. **b** Analysis with the substrate L-Tyrosine: **P < 0.01, 1 dpi in comparison with the control; solid arrow (*P* < 0.05), 13 dpi in comparison with 1 dpi. **c** Analysis with the substrate PPD; *P < 0.05 and ***P < 0.001, 1 dpi, 8 dpi, and 13 dpi in comparison with the control; solid arrow (*P* < 0.001), 13 dpi in comparison with 1 dpi and 8 dpi; ## *P* < 0.01, 30 dpi in comparison with 13 dpi

We observed PO activity with the substrates L-DOPA, L-tyrosine, and PPD. Figure 1 illustrates PO concentration in the haemolymph of *A. fulica* infected with *A. vasorum* and controls.

PO levels with all the substrates peaked at 1 dpi, in comparison with the control, using the substrates L-DOPA (*F*_(4,90)_ = 22.67, *P* = 0.02), L-tyrosine (*F*_(4,90)_ = 6.73, *P* = 0.00006) and PPD (*F*_(4,90)_ = 27.58, *P* = 0.0019) was observed (Fig. 1a, c).With activity reduction at 8 dpi for the substrates L-DOPA and L-tyrosine (2.0 and 1.55 times lower than that observed at 1 dpi, respectively) while activity with PPD remained similar. And PO activity control compared to L-DOPA (*F*_(4,90)_ = 22.67, *P* = 0.00002) decreased, while in PPD (*F*_(4,90)_ = 27.58, *P* = 0.079) increased (Fig. 1a and c). At 13 dpi, PO levels with the substrates L-DOPA and L-tyrosine remained stable, while activity with PPD decreased (1.73 times lower than that observed at 8 dpi) and PO decreased in L-DOPA (*F*_(4,90)_ = 22.67, *P* = 0.00029) and PPD (*F*_(4,90)_ = 27.58, *P* = 0.000015) in relation to control (Fig. 1a and c). At 30 dpi, PO levels increased with the substrates L-DOPA and PPD (1.63 times higher than at 13 dpi) and activity with L-tyrosine remained constant, there was no statistical difference in relation to the control (Fig. 1a and c). Activity with the substrate PPD was the highest in the cell-free haemolymph at all times analyzed.

The populations of haemocytes showed 95% viability before sonication, and lysate haemocytes of *A. fulica* showed no PO activity.

### NO levels in *Achatina fulica* haemolymph during parasite infection

**Fig. 2:**
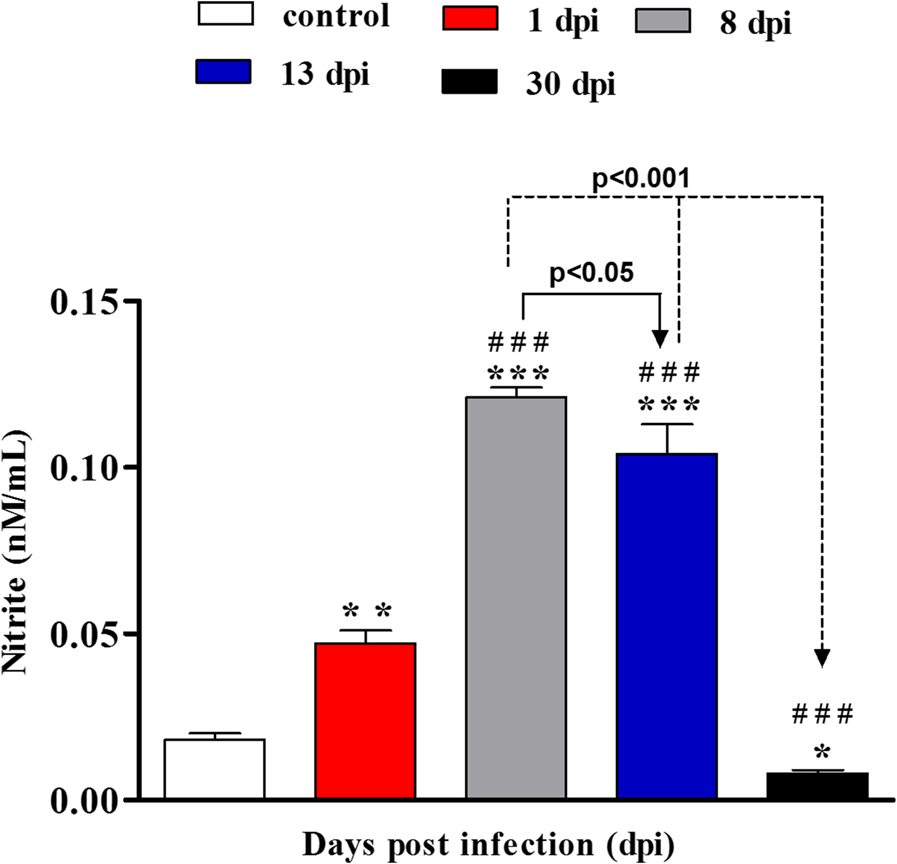
Nitrite contents in the soluble fraction of the haemolymph of *Achatina fulica* during *Angiostrongylus vasorum* infection. Each *column* represents mean and standard deviation of the nitrite in haemolymph. **P < 0.001, ***P < 0.001 and *P < 0.05 represent the statistical significance of 1 dpi, 8 dpi, 13 dpi, and 30 dpi, respectively, in comparison with the control; ### *P* < 0.001, 8 dpi, 13 dpi, and 30 dpi in comparison with 1 dpi; arrows (*P* < 0.05 and *P* < 0.001), 13 dpi and 30 dpi, respectively, in comparison with 8 dpi

NO levels were indirectly estimated in the soluble fraction of *A. fulica* haemolymph through the measurement of NO_2_ (Fig. 2). Until 13 dpi, the non-infected molluscs showed lower amounts of NO in the haemolymph (0.018 nM) in comparison with those infected; but at 30 dpi, NO levels were lower than those observed for controls. The peak of NO was registered at 8 dpi (0.12 nM) and was 6.5 times higher than the control (*F*_(4,24)_ = 94.59, *P* = 0.003687). The amount of NO started to decrease at 13 dpi but was still significantly higher than the control (*F*_(4,24)_ = 94.59, *P* = 0.001). At 30 dpi, NO levels were 15 times lower than the levels observed at 8 dpi (peak).

### Histology

**Fig. 3:**
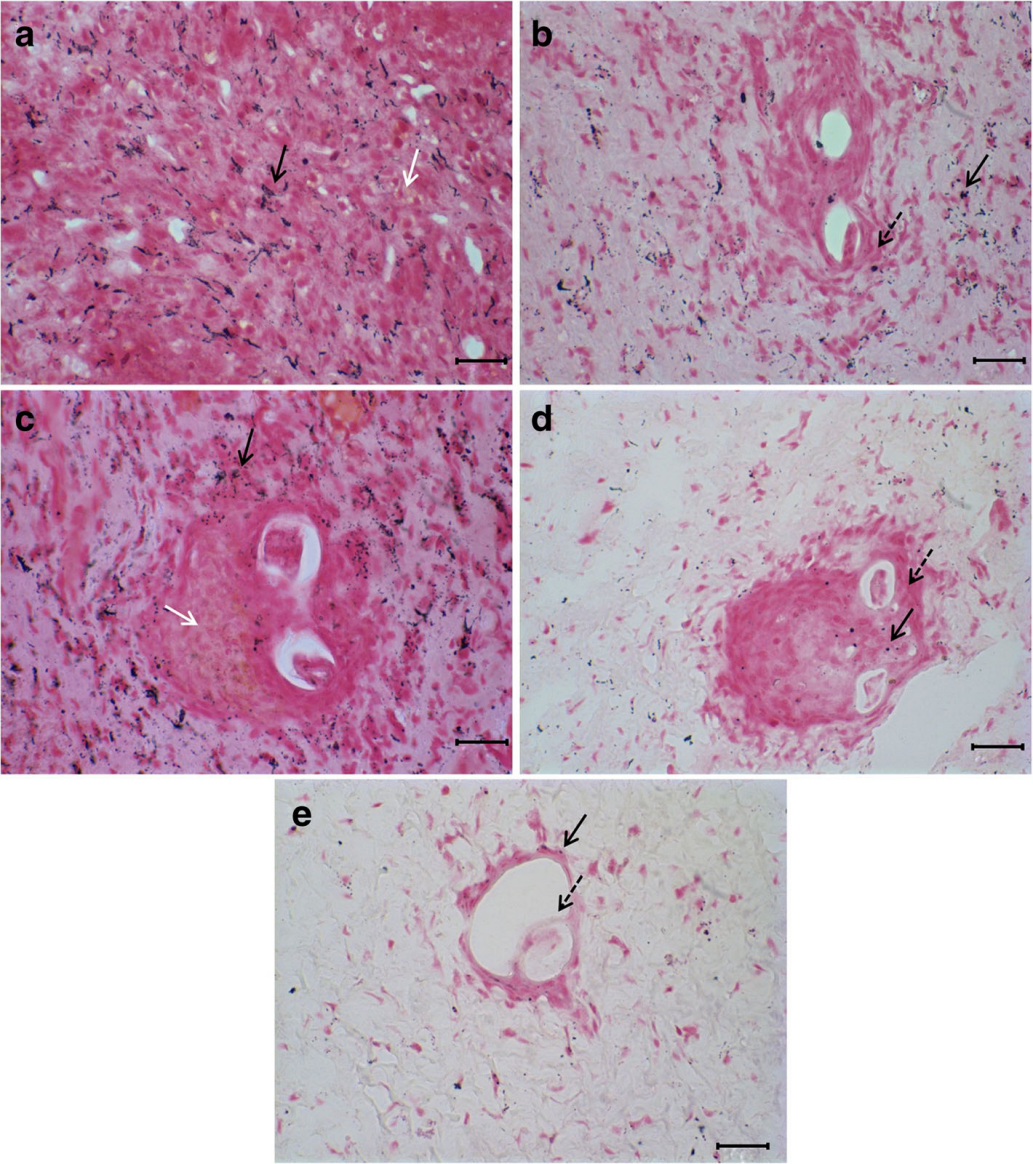
Histological sections of the cephalopodal region of *Achatina fulica*. **a** Control, melanin deposition (black arrow) and melanin production (white arrow) in the tissue. **b** 1 dpi, L1 surrounded by cellular infiltrate (dashed arrow) with the presence of melanin (black arrow). **c** 8 dpi, the presence of melanin (black arrow) and melanin production (white arrow). **d** 13 dpi, larvae involved in cellular infiltrate (dashed arrow), with a low amount of melanin (black arrow) in the surroundings. **e** 30 dpi, larvae involved by less dense cellular infiltrate (dashed arrow) and low amount of melanin in the surroundings (black arrow). *Scale-bars*: 25 μm

The melanin presence in the cephalopod tissue of *A. fulica* in molluscs of the control (Fig. 3a) and infected groups (Fig. 3b-e) was confirmed performing Fontana-Masson staining, which is specific for melanin. At 1 dpi a cellular infiltrate surrounding the *A. vasorum* larvae with melanin deposition was observed (Fig. 3b). In this tissue, L1 were observed wrapped by the cellular infiltrate and a discrete presence of melanin in the surroundings (Fig. 3b). At 8 dpi, the cellular infiltrates increased around the *A. vasorum* larvae, and the L2 deposition of melanin in the tissues seemed to be more pronounced in comparison with what was observed at 1 dpi (Fig. 3c). At 13 dpi, when L2 become L3, a reduction in melanin deposition on the tissue and surrounding the larvae occurred (Fig. 3d). We observed a decrease of the cellular infiltrate surrounding L3 *A. vasorum* at 30 dpi, and on this day, the melanin deposition decreased comparing to the other days of infection (Fig. 3e).

**Fig. 4:**
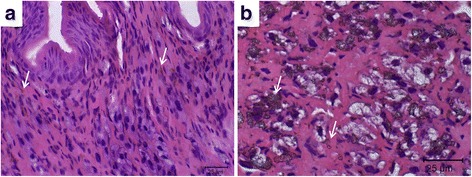
Histological sections of the cephalopodal region of *Achatina fulica*. **a** Melanisation spots in the tissue of *Achatina fulica* control (white arrows). **b** Tissue at 1 dpi, presence of melanin crystals in tissue (white arrows). *Scale-bars*: 25 μm

Through hematoxylin-eosin staining allowed to verify the presence of melanin on the outside of the cells in the infiltrate (Fig. 4a). The presence of melanin crystals in the tissue on every analyzed day of this study, as represented at 1 dpi (Fig. 4b), was confirmed.

## Discussion

In the present study, we demonstrated that the soluble fractions of the haemolymph of *A. fulica* had PO activity and showed the presence of melanin in the cephalopod tissue; however, snails infected with *A. vasorum* showed higher PO activity on some days. PO enzymatic activity was detected both with the non-specific substrate L-DOPA, which metabolizes catecholase, laccase and tyrosinase, and the substrates specific for laccase (PPD) and tyrosinase (L-tyrosine).

Very few studies have explored PO activity in molluscs, and most models involving molluscs do not have PO activity with the substrate L-tyrosine [15, 34]. Activity with this specific substrate is frequently observed in insects such as *Drosophila melanogaster* [14, 35, 36]. Here, we demonstrated mollusc reactivity to the substrate L-tyrosine, and melanin was circulating in the haemolymph spaces, surrounding the parasites, as observed in the melanotic capsules of insects. The staining intensity of the melanin deposition, however, differed from that observed in insects [20, 37, 38]. Unlike some populations of insects [36], melanin of *A. fulica* was not detected inside the circulating haemocytes, nor in the cells infiltrated in the tissues analyzed.

PO activity in the haemolymph at 1 dpi, detected with the substrates L-DOPA, L-tyrosine and PPD, coincided with the beginning of the detection of melanin in the epithelium. However, a lower amount of melanin was observed in the connective tissue in the surroundings of L1, where cells migrated.

At 8 dpi, the levels of PO detected in the haemolymph decreased with L-DOPA and L-tyrosine substrates, in this day the moult from L1 to L2 may modulate PO activity through the antigens produced during the moult. The melanin and the cellular infiltrate increased in the connective tissue surrounding L2, suggesting that this tissue could lead to a mechanical contention of the parasite.

Melanin circulates in the tissue infiltrate through the haemolymph spaces and, during the activation of PO pathway in insects, cytotoxic elements such as quinones and oxygen and nitrogen derivates are produced to boost the destruction of the pathogens’ tegument [20, 39, 40]. We suggest that a similar mechanism may occur in our model, as *A. fulica* presented the highest levels of NO in the haemolymph at 8 dpi.

The transformation from L2 to L3 seems to also negatively interfere with PO activity at 13 dpi for all the three substrates tested, and particularly PPD. At this interval, the levels of NO and circulating melanin in the epithelial and connective tissues and the cellular infiltrate next to the parasite started to decrease.

Finally, at 30 dpi, the modulation of the L-tyrosine-dependent PO pathway continued at the same level as the previous days of infection, whereas PO levels increased with L-DOPA and PPD. The PO oscillations observed during larval development may occur due to the detection of products released by L1 and L3 when *A. vasorum* penetrates and exits the *A. fulica* tissues [41, 42]. Another factor to be considered is the activation of PO pathway in the nematode cuticle as described by Brivio et al. [43]. In insect *Diatraea flavipennella* infected with the parasitoid *Cotesia flavipes*, modulation was observed in NO and PO levels throughout parasite infection [20]. However, further studies are needed to a better understand this mechanism.

The decrease of NO at 30 dpi in comparison to the control may be due to the association of biological characteristics of L3 of *Angiostrongylus* spp. that exhibits motility and allows its active output from the granuloma in molluscs tissues [44, 45]. Also, L3 eliminating excretory/secretory products may contribute to increase or decrease the activation levels of the innate immune defence system of molluscs [42, 46]. In *Biomphalaria tenagophila* infected with *A. vasorum* at 30 dpi, a decrease in NO level was observed in relation to the control group [8].

Here, we suggest that *A. fulica* presents an innate immune defence mechanism based on PO pathway. According to Allam & Raftos [47], this mechanism could be important for pathogen recognition. At 1 dpi, when the melanin in the tissue close to the parasite is still unremarkable, cytotoxic products, such as NO, start to increase in the soluble fractions of the haemolymph, thus indicating that recognition of *A. vasorum* may be taking place. Le Clec’h et al. [15] observed that *Biomphalaria glabrata* infected with *Schistosoma mansoni* presents laccase-dependent PO activity but did not associate the activation of this pathway with the susceptibility to the parasite. The oscillations in the levels of PO, tissue melanin, and NO observed in our model may contribute to the control of the infection. Indeed, Coaglio et al. [6] recovered only 8% of L3 in *A. fulica* infected with *A. vasorum*, in contrast with the over 80% recovery of L3 reported by Mozzer et al. [3] and Barçante et al. [45] in *Omalonyx matheroni* and *B. glabrata*.

Unlike the arthropods (insects and crustaceans) that have PO located mainly in the haemocytes, we did not detect a response of PO in the populations of haemocytes of *A. fulica* during the infection with *A. vasorum,* similar to previous observations in *B. glabrata* [15] and in haemocytes of *Lymnaea stagnalis* infected with *Plagiorchis* sp. [34]. In insects such as *D. melanogaster*, PO is located in the crystal cells and lamellocytes [36, 48].

L-tyrosine-dependent PO activity is frequent in arthropods [36, 49]. To the best of our knowledge, we show herein for the first time, its presence in the soluble fractions of the haemolymph of *A. fulica*, which may explain the lower susceptibility of *A. fulica* to *Angiostrongylus* spp. [6, 50, 51].

## Conclusions

We suggest that the cytotoxic products, such as NO, released in the PO pathway, may play a role in controlling the infection inside the mollusc. Further studies will be needed to understand the PO-dependent defence mechanisms in this terrestrial mollusc.

## Abbreviations

dpi: day(s) post-infection
L1: first-stage larvae
L2: second-stage larvae
L3: third-stage larvae
LPS: lipopolysaccharides
NO: nitric oxide
PO: phenoloxidase
PPD: p-phenylenediamine
proPO: phenoloxidase inactive form
RNS: species reactive nitrogen
ROS: species reactive oxygen
